# Flood Risk and Preventive Choices: A Framework for Studying Human Behaviors

**DOI:** 10.3390/bs14010074

**Published:** 2024-01-20

**Authors:** Alessandro Sapienza, Rino Falcone

**Affiliations:** Institute of Cognitive Sciences and Technologies, National Research Council of Italy (ISTC-CNR), 00185 Rome, Italy; rino.falcone@istc.cnr.it

**Keywords:** floods, hydrogeological phenomena, natural risk, climate change, social simulation, agent-based framework, cognitive modeling

## Abstract

The topic of flood phenomena has always been of considerable importance due to the high risks it entails, both in terms of potential economic and social damage and the jeopardizing of human lives themselves. The spread of climate change is making this topic even more relevant. This work aims to contribute to evaluating the role that human factors can play in responding to critical hydrogeological phenomena. In particular, we introduce an agent-based platform for analyzing social behaviors in these critical situations. In our experiments, we simulate a population that is faced with the risk of a potentially catastrophic event. In this scenario, citizens (modeled through cognitive agents) must assess the risk they face by relying on their sources of information and mutual trust, enabling them to respond effectively. Specifically, our contributions include (1) an analysis of some behavioral profiles of citizens and authorities; (2) the identification of the “dissonance between evaluation and action” effect, wherein an individual may behave differently from what their information sources suggest, despite having full trust in them in situations of particular risk; (3) the possibility of using the social structure as a “social risk absorber”, enabling support for a higher level of risk. While the results obtained at this level of abstraction are not exhaustive, they identify phenomena that can occur in real-world scenarios and can be useful in defining general guidelines.

## 1. Introduction

Critical weather phenomena and, in particular, floods represent a serious inconvenience for all governments due to the damage they can cause, both in terms of human lives (a single flood could cause the deaths of millions of people [1,2]), which is the primary reason why governments should seriously care about floods and the huge economic losses they incur.

Data concerning this problem are definitely alarming. Cuñado and Ferreira [3] report that floods represented 40% of all natural disasters between 1985 and 2009; Luino and colleagues [4] report that, in Italy, “In the 20-year period from 1980 to 2000 the State set aside 7400 million euro for flood damage, or roughly one million euro per day”; Guha-Sapir and colleagues [5] state that, in 2013, hydrological disasters took the largest share of natural disaster occurrence (48.2%) and that the most expensive hydrological disaster ever registered happened in Thailand in 2011, causing USD 41.4 billion worth of damages.

These types of events are expected to become increasingly emphasized due to climate change [6,7]. Indeed, we expect severe weather events to increase in severity and frequency [8,9]. For instance, it has been predicted [10] that there will be a mean increase in the 100-year precipitation event by 20% in magnitude and >200% in frequency in a high-warming scenario.

Nevertheless, assessing the comprehensive adverse consequences of a natural disaster is a challenging task. The types of damage we have just discussed pertain to direct harm inflicted upon buildings, infrastructure, residences, roads, and agriculture. Beyond these readily visible and quantifiable direct damages, natural disasters also involve a multitude of secondary and indirect consequences that pose substantial challenges in terms of detection and assessment. For instance, being subjected to a natural disaster could lead to a decrease in tourist flows, resulting in turn in a worker’s dismissal. An exemplary case is that of Umbria, a central region of Italy that, in 2016, was repeatedly struck by earthquakes. The entire area reported extensive damage to streets, houses, and structures in general. But this is not all; the population is still paying the consequences. In fact, once people start perceiving a particular geographical area as dangerous, it is not easy to change their minds. As the result, the local authority reported in its data [11] that tourism, representing one of the most important sources of earnings for the local population, decreased by 35%.

Similarly, the recent flood in Emilia Romagna [12] resulted in significant losses in terms of casualties and direct damage to structures and infrastructure. In this case as well, there was a significant decline in tourism, with some provinces experiencing a reduction of 20% in local tourism and even 27.8% in foreign tourism [13]. Moreover, psychological traumas [14,15,16] or other kinds of mental disease [17] may arise after a disaster due to the risk of dying or to the loss of material goods or even someone we care about.

From the above considerations, it becomes evident that there is a need to identify more effective ways to control the impacts of floods and mitigate the damages they cause. Indeed, as [18] reports, “self-protective behavior by residents of flood-prone urban areas can reduce monetary flood damage by 80%, and reduce the need for public risk management”. Then, while it is indeed true that floods pose a significant threat to both authorities and the general population, it is also true that citizens’ adoption of self-protective measures can greatly mitigate the issue of both direct and indirect damages. Therefore, on the one hand, it is necessary to investigate in detail the decision-making processes of citizens, and, on the other hand, it is also crucial to identify community policies and improve strategic planning to reduce the impacts of critical hydrogeological phenomena. This is precisely the task of national governments and local authorities, which play a key role in this context [19,20].

We support the thesis that authorities should identify effective strategies to foster positive behaviors within populations. Their role extends beyond merely providing assistance after a disaster; they should also have the goal of promoting preventive behaviors and actions among the populace, which can in turn minimize future risks. In conclusion, the role of the authority within this framework should be as follows:

1. To promptly inform the population with the most reliable forecast about what is going to happen [21].
2. To provide information properly: for instance, the authors of [22] analyze community resilience, i.e., the capacity of a community to lead itself in order to overcome changes and crises. They showed that this value positively correlates with community satisfaction concerning the information that the authority reports.
3. To encourage citizens to undertake self-protective behaviors as they can substantially reduce the problem of direct and indirect damages [23,24].

The damage a population suffers strictly depends on the relationship between the citizens and the authority, from their collaboration and coordination to their reciprocal sensitization towards a safety culture. Citizens use a strategy to estimate the risk and decide how to behave. Even though the authority possesses its own strategy, it could have a different purpose; more precisely, each citizen wants to protect themselves while the authority needs to protect and safeguard the collective interest. In general, there should be a convergence between these two interests. Nevertheless, there may be important differences. As a consequence of the contradicting goals between citizens and the authority, a behavioral study of these actors becomes essential to understand how they relate and what the outcome of their interaction is.

In this work, we propose a multi-agent system approach, creating a social simulation in which the citizens and the authority are modeled through cognitive agents. We intend to provide a useful tool to analyze the problem both from the authority’s and from the citizens’ point of view. By means of simulations, we investigate the authority’s role and the possible choices it has to provoke the expected citizens’ behavior. The citizens have, in turn, their own strategies to detect the most appropriate behavior in relation to real events and the sources (including the authority) predicting them. The model is designed to investigate the evolution of decision-making strategies by citizens and authorities during floods. Therefore, the model simulates the following:

1. The pre-flood phase, where strategies are implemented to face the event.
2. The evolution of the event.
3. The damages caused by the event.

It is intended to not only assess the impact of an event on a population but also, above all, to evaluate the effects of authorities’ strategies in the pre-flood phase.

In more detail, the objectives that this work aims to achieve are as follows:

1. Development of a social simulation platform based on multi-agent systems to explore community behavior in the face of critical hydrogeological phenomena.
2. Analysis of citizens’ decision-making processes in hydrogeological risk situations, considering subjective risk perception and their response to external information and stimuli.
3. Evaluation of the strategies available to the authority to encourage citizens to adopt appropriate behaviors in the face of hydrogeological risks, considering both incentivizing and punitive approaches.
4. Exploration of emerging social dynamics between citizens and authorities in risk contexts.
5. Study of the role of institutional trust in this context, examining how trust influences the effectiveness of strategies adopted by authorities.

Overall, the main goal is to contribute to a better understanding of the interactions between citizens and authorities in hydrogeological risk contexts and to provide useful tools to address these challenges more effectively.

The rest of this article is organized as follows. In Section 2, we describe related works. In Section 3, we introduce the trust model used to handle information sources. Section 4 is dedicated to the realized platform. In Section 5, we analyze two possible scenarios. In conclusion, Section 6 summarizes the results of the whole work.

## 2. State of the Art

The current literature mainly focuses on identifying and quantifying the damage that weather phenomena and floods may cause. Fewer studies focus on the population’s response. If we extend the search field to other types of natural risks, such as bushfires [25] or earthquakes [26], there would be other studies to consider. Unfortunately, it is not possible to use their conclusions and findings since the risk attitude is domain-dependent [27], so their results cannot be applied to floods. It is, however, interesting to see how these works approach the problem.

### 2.1. Quantifying Damages

A possible way to deal with this topic is to take into account the historical evolution of the phenomena that happened in a given geographical area: the way they happened, their intensity, frequency, etc. For instance, [28] analyzed all the historical natural events that occurred in the area of Valtellina di Tirano, a mountain area in the Central Italian Alps, to produce a georeferenced database. The study demonstrates the significance of collecting and leveraging historical information, emphasizing its crucial role in identifying potential critical scenarios, assessing territorial threats, and effectively managing future emergencies.

A second line of contribution exploits the simulation approach to estimate the damage an event can cause [4,29]. For instance, in [4], the authors propose a model simulating critical scenarios and evaluating the expected economic loss. Here, the flood water level is considered as the factor indicating the event magnitude, which is a questionable simplification (for instance, Grahn [30] states that including the duration of the event is a key factor). Another interesting contribution is that of Tierolf and colleagues [31]. As urban development is concentrated in river areas in Asian countries, there is a risk that this intense urbanization may increase flood risk. Therefore, the authors focus on quantifying the impact of anthropization, projecting flood risk until 2040, and estimating the potential increase, which in some cases is up to +211%.

Please note that there is no consensus in the literature on how the magnitude of events should be classified. We refer to that produced by [32]. Here, the damage due to natural disasters is divided into four types. They mainly distinguish between *tangible* (monetarily quantifiable) and *intangible damage*, which is more difficult to quantify (such as loss of life, psychological traumas, etc.). In turn, these are classified into *direct*, that is, the damage caused directly by the event (damage to roads, buildings, houses, etc.), and *indirect*, i.e., the secondary damage that the event causes, such as the closure of companies, the decline in tourism, etc. Usually, researchers focus on tangible direct damage since this is the most practical dimension to quantify economically. When a critical event affects a given area, the proposed model computes the economic loss due to direct damage as a function of (1) the economic value of the exposed area and (2) the degree of damage. The necessary condition for the model to produce the output is therefore that there is a good knowledge of the local area and a description of the physical event.

### 2.2. Risk Perception and Population’s Response

In this subsection, we will now discuss the specific approaches available to quantify risk perception and to identify the reactions of citizens in the presence of floods. The study of risk perception concerns the analysis of people’s knowledge and, consequently, the study of their behavior concerning hazards.

The first and most common methodology is that of a survey [33,34,35], which aims to identify what people did in the past or what they would do while facing a critical flood. Some research relies on socio-demographic factors [36], while others state that psychological factors provide a better picture of the situation [18]. Usually, the authors start by proposing a psychological model, and then they lean on surveys to identify the correlations among their variables and a regression analysis to try to explain people’s behavior. Among these, Cao and colleagues [37] investigate the factors having a significant impact on protective coping behaviors. Among the main factors, trust in the government was found to be positively related to protective coping behaviors. Surveys represent a useful instrument; however, sometimes what people say does not correspond to what they actually do [38].

At last, social simulation can be used to model the choice and the behavior of a population during floods. The purpose of this method is to provide a tool for emergency managers, as it can lead to higher-quality decisions and a higher level of emergency preparedness. Simonovic and Ahmad [39] propose a computational framework where the human decision-making process is modeled following the work of [40]. The authors identify the mathematical relations between the different variables of the model, implementing them in a simulation framework. Among the most important variables they identify for determining the final decision, we find their previous flood experience, the impact of the warning, and the behavior of the others.

In a similar way, even though they refer to bushfires, Adam and Gaudou [25] propose a multi-agent system, modeling fires, houses, shelters, and residents as agents. Their study aims to understand the population’s behavior in response to various communication strategies during bushfires.

In the domain of multi-agent simulation, an interesting contribution is provided by Tonn and colleagues [41]. The authors introduce a model to simulate how individual behavior influences flood risk over time in future climate scenarios. Although the purpose of the simulation is exactly opposite to ours, it is certainly a highly relevant topic. Furthermore, the results are closely tied to the specific context under consideration: the city of Fargo, ND, USA.

Another remarkable study by Dawson and colleagues [42] introduced a quantified modeling strategy to predict the probable exposure of individuals to flooding across various storm surge conditions. The study extensively examined scenarios involving breaches in defenses, different flood warning durations, and various evacuation strategies. Within the suggested ABM framework, interactions and feedback between floods and human responses were simulated dynamically as the event unfolded. Notably, a probabilistic finite state machine was employed to characterize the behaviors of agents, encompassing potential states, permissible actions, and transitions between states.

In Li et al. [43], the authors explore the simulation of flood evacuation, a complex geographic process involving the dynamic attributes of floods, field patches, and their interactions with crowd behaviors. They introduce a Cellular Automata and Multi-Agent System (CA-MAS) model to integrate water evolution, land cover, objective domain, crowd, and individual movement data. The simulation experiments indicate that the weighted potential field method is a practical approach to assessing individuals’ movement inclination. The study is regarded as initial research and an illustration of the CA-MAS model, with future considerations for different flood levels, evacuation plans, and individual attributes. The authors propose using augmented reality technology for real-participant flood evacuation experiments in virtual environments to validate simulation models realistically.

Thus, both works give importance to the communicative aspect in determining citizens’ behavior. Moreover, they both use surveys to identify which parameters characterize the population. While this choice is optimal for modeling a specific population, it is well established in the literature that many of these parameters, such as risk perception [44], the potential damage they may incur, and the trust they place in their information sources [45], vary from one population to another and from one context to another. Therefore, such data cannot be generalized, and the results under consideration are strongly tied to the specific population under analysis. On the other hand, a generalizable approach requires the identification of behavioral patterns of the involved agents while keeping the setting of variables that are linked to the specific population parametric. Therefore, to be reusable, the platform needs to be parametric. By doing so, this approach enables emergency managers and local authorities to later utilize the model by simply specifying the characteristics of their own population within the framework. The advantage of this approach is evident: it allows for the exploration of various potential scenarios before an actual crisis occurs, with the flexibility to adjust input parameters as needed.

Additionally, the contributions we analyze make use of two different approaches to simulate human behavior. The first one is equation-based, exploiting mathematical equations and functions to model behaviors and the overall state of the system. The second one, the agent-based model, is particularly suited for the representation of human behavior and for the investigation of complex phenomena emerging from the interactions of individual agents, especially if the population is composed of heterogeneous individuals [46,47]. In both cases, the overall state of the system is a consequence of the individual agents’ actions.

In the context of a literature review, a notable scarcity of studies on behavioral aspects regarding population responses to natural disasters, with a specific focus on floods, becomes apparent. This lack is particularly relevant when considering that findings from studies on other natural phenomena cannot be directly applied to floods due to the context-dependent nature of risk behavior [27]. Our research addresses a significant gap in the current literature, which predominantly centers on the identification and quantification of damages caused by meteorological events and floods. What makes our contribution relevant is the in-depth exploration of the behavioral aspect of the population in response to floods; an area that has received comparatively less attention. This includes an analysis of coping strategies, with particular attention to the citizen–authority relationship. Moreover, since our aim is to focus on behavioral dynamics rather than the specifics of the particular scenario under consideration, the developed platform is not limited to modeling a specific population or region. Therefore, it can be easily generalized to other study contexts, as long as the social dynamics among interacting entities remain consistent.

## 3. Managing Information through Trust

The literature [48] demonstrates how trust, or the lack thereof, in authorities is one of the most important factors in risk perception regarding natural hazards. For the management and operationalization of the concept of trust, as well as its relative use in managing information sources, we will refer to the socio-cognitive model of trust [49]. Indeed, in addition to being one of the primary cognitive models of trust, it is of particular relevance in this context as it allows us to articulate trust based on its sub-components. According to [49], trusting an information source (S) means using a cognitive model based on the dimensions of competence and reliability/motivation of the source, which in turn may arise from different reasons [50]:

1. Our previous *direct experience* with S on that specific kind of information content.
2. *Recommendations* (other individuals reporting their direct experience with and evaluation of S) or *reputation* (the shared general opinion of others about S) on that specific information content [51,52,53,54,55].
3. *Categorization* of S (it is assumed that a source can be categorized and that its category is known), exploiting inference and reasoning (analogy, inheritance, etc.): on this basis, it is possible to establish the competence/reliability of S on that specific information content [56,57,58,59].

The trust model used in this work exploits the trust definition defined in [49] and mainly relies on direct experience to produce evaluations. Indeed, we also use categorization analysis to distinguish the sources based on their different natures. Trust decisions in the presence of uncertainty can be handled using uncertainty theory [60] or probability theory. We decided to use the second approach since, on this platform, our agents work with probabilities, possessing an a priori knowledge of all the possible events that can occur and are able to estimate how plausible it is that they occur. We decided to exploit Bayesian theory [61], one of the most used approaches in trust evaluation [62,63,64], and we based our concept of trust on [49].

This model is composed of a set of citizens *C*, sources *S*, and information *I*. Each citizen $c_{k}\in C$ evaluates the performance of its information sources to understand how trustworthy they are concerning a specific kind of informative task. They will use the function *trustOnSource: S* × I→T, where *T* is a real value defined in the range [0,1], producing a different evaluation for each source according to their direct experience.

Initially, there is no evidence about how trustworthy these sources are; thus, the initial evaluations are set to 0.5, a value describing a situation of substantial uncertainty between trust and distrust. Each source $s_{j}\in S$ possesses the information $i_{j}\in I$, represented by a probability density function (PDF). This PDF is divided into three segments, corresponding to the intensity of the event (light, medium, and critical). The citizens can access the PDF through the function *getInfo: S* × T→I. Here, the trust value is fundamental. In fact, by means of the getInfo function, information reported by the sources is properly manipulated according to trust. The trust evaluation that the citizen attributes to the source is used to appropriately weigh the informative contribution of the source, in the form of a PDF, compared to the global evidence that they possess. The idea is that the agent trusts what the source $s_{j}$ reports proportionally to how much they trust the source itself (as a source of information). The individual segments of the PDF are processed according to Equations (1) and (2):

(1) $$segment_{k}^{smoothed}=\left(1+\left(segment_{k}-1\right)*trustOnSource\left(s_{j},i\right)\right)$$

(2) $$segment_{k}^{norm}=\frac{segment_{k}^{smoothed}*N}{\sum_{k=1}^{N}segment_{k}^{smoothed}}$$

Equation (2) has the sole purpose of normalizing the PDF (in order to be a PDF, its area needs to be equal to (1)). Here, *N* is the number of segments and it is equal to 3; thus, the index *k* goes from 1 to *N*. The output $segment_{k}^{smoothed}$ of Equation (1) is the update of the segment, but it still needs to be normalized. This is performed in Equation (2), which in fact takes as input $segment_{k}^{smoothed}$ to produce $segment_{k}^{norm}$.

Once the PDF for each information source is estimated, there is an aggregation process aggregate: I×I→I. Each agent initially possesses a flat global distribution (global PDF or GPDF) as there is no evidence/information. Then, they will add the information coming from each source. To this purpose, it is possible to use the classical Bayesian logic recursively on each source:

(3) $$\begin{array}{c} GE^{updated}=aggregate\left(GE,i\right)=\frac{GE*i}{NF} \end{array}$$

In Equation (3), NF is a normalization factor that, as for *i*, ensures GE is still a probability density function. In other words, $GE^{updated}$, that is, the global evidence that an agent has, is computed as the product of the old GE and the new contribution reported by the source. The probability that each event will happen is obtained by integrating information *i* in the segment representing the specific event. The citizens will reason about these probabilities to make their decisions. Since we consider three events, the PDFs are split into three parts.

### 3.1. Updating Trust

Our citizens adapt to the world they live in, which, within this framework, means updating their trust assessments [65,66] towards information sources in order to precisely understand how reliable their sources are. At the beginning, they solely possess a neutral trust value, which will then be updated based on direct experience found around the world. The new trust value ($t_{kj}$: from the agent *k* to the agent *j*) is computed as the weighted mean of the old evaluation and the new performance:

(4) $$\begin{array}{c} t_{kj}^{updated}=\frac{\alpha *t_{kj}+\beta *newPerformance_{j}}{\alpha +\beta } \end{array}$$

In Equation (4), α and β represent, respectively, the weights of the old trust value and the newly realized experience. It is clear that they play a critical role, so it is important to define them properly. We fixed the value of α to 10; on the contrary, β changes in the range [0,10] based on what the source reported and what actually happened. In this way, we can ensure that the weight of the source’s performance on trust is proportional to the impact that its information had on the agent. In other words, the greater the criticality of the task (the risk to which the agent is subjected, the effective damage, and the cost of the agent’s decision IncurredCost) the greater will be β. In particular, if the source suggests investing (for critical and medium events), we will consider AvoidedDamage, the damage the agent avoided thanks to the source (or that it would have avoided if it had listened to the source), as in Equation (5), while for light events’ forecasts, we will consider the incurred damage IncurredDamage, as in Equation (6). In both cases, we will take into account the decision cost:

(5) $$\begin{array}{c} \beta =|AvoidedDamage-IncurredCost| \end{array}$$

(6) $$\begin{array}{c} \beta =|IncurredDamage-IncurredCost| \end{array}$$

In Equation (4), $t_{k}j$ is the previous trust degree and newPerformance is the objective evaluation of the source performance. This last value is obtained by comparing what the source said with what actually happened. We take into account just a portion of the reported PDF, i.e., the estimated probability of the event that actually occurred.

## 4. Materials and Methods

In this work, we propose a social simulation tool to study how a population subjected to the risk of a critical weather event behaves, based on different risk estimation methods and external inputs. In fact, in our perspective, we do not focus only on the decision-making process but also on the information sources the population can use to evaluate the risk. This is why we decided to use a multi-agent system to study the problem: it models the presence of different (cognitive) agents possessing different goals and reasoning about the situation to make the best choice. This approach to the problem allows us to analyze the intriguing social phenomena that arise, as we can model agents in the world starting from their specific behaviors and study how their complex interactions give rise to emerging social phenomena. Furthermore, this approach provides us with the opportunity to examine the role and influence of individual actors and parameters in shaping the final decisions of citizens.

In our specific case, we are interested in studying the response of a population distributed in a particular geographic area to various hydrogeological phenomena of different criticality and danger levels. Individual citizens aim to stay safe, and to achieve this, they need to gather information, reason, and act in the most appropriate manner possible. In this regard, the choice to model citizens as agents and to refer to multi-agent systems is not only natural but also essential, as it allows for the modeling and representation of all these concepts.

We realized the simulation using NetLogo [67] and implemented the trust model (see Section 3) in Java as an extension to the predefined NetLogo framework.

When the simulation starts, the simulation world is populated by several cognitive agents (citizens) that are randomly distributed and have the need to identify the future weather event based on the information sources they have and the trustworthiness they attribute to these different sources. To cope with these events, citizens possess an initial capital they must manage by making the right investments. Therefore, they need to determine which choice is most advantageous for them based on the potential costs and damages associated with each decision. In addition to citizens, there is another agent called the authority. Its objective is to promptly inform citizens about weather phenomena, ensuring that they act promptly and in the best possible way to address the event.

When an event occurs, it can cause huge damage to citizens’ personal capital and to the authorities’ social capital; thus, it is fundamental for them to understand what to do.

### 4.1. Severity of Flood Phenomena

The possible phenomena are categorized using, as a starting point, the classification provided in [28], which is based on the provoked damage. They distinguish events as *high*, affecting people and buildings; *medium*, affecting infrastructure; *low*, affecting agricultural and forest areas; and *no damage*. To simplify, we associated low with no damage, so we considered them as the same event. Consequently, we implemented in the framework three possible events: 1 (light or no event), 2 (medium event), and 3 (critical event). Each of these events can cause damage to citizens’ personal capital and to the authority’s social capital.

Note that the events we are going to analyze happen in a window covering a long period of time (months); we are not analyzing short-term situations.

### 4.2. Information Sources

In this article, we focus on sources of information rather than channels of information. In this regard, we refer to information producers (authorities, citizens through direct observation of the phenomenon, citizens through observation of others’ behavior) rather than information communication means (TV, radio, social media, etc.). What we consider are categories of sources, which can therefore have multiple instances. Initially, each citizen possesses a given amount of knowledge about critical weather phenomena. This knowledge can derive from different factors, such as past experiences or environmental or other external factors, and it could be influenced or biased by social or psychological factors. In addition to this knowledge, the citizens can consult a set of different information sources reporting evidence about the incoming meteorological phenomenon. Given that all the information the citizens possess and that the sources report concerns probabilities, we decided to represent it as a probability density function, or PDF.

In particular, we considered three kinds of sources (whether active or passive) in our framework:

1. Citizens’ *personal judgment*, based on their direct observation of the phenomena. Actually, a common citizen is usually not able to understand the situation, which may be because they are not skillful, do not possess the skills needed for the given condition, or do not possess any instrument to conduct a proper evaluation.
2. *The authority* disseminates weather forecasts around the world, aiming to provide citizens with information about upcoming events.
3. Observation of *others’ behavior* (other agents in the radius of three NetLogo patches): agents are somehow influenced by community logic, tending to partially or totally emulate their neighbors’ behavior. In the social source’s PDF, each event has a probability directly proportional to the number of neighbors making the related decision. This source can have a positive influence if the neighbors behave correctly; otherwise, it represents a drawback.

It is worth underlining that we take care to distinguish assessments made through direct experience from those made through appropriate instruments and models for predicting meteorological phenomena. Direct observation of the phenomenon is a useful means for citizens to understand the level of hydrogeological risk, but it has intrinsic limitations. Firstly, the majority of citizens are not experts in hydrogeological phenomena and may therefore be unable to correctly interpret complex signals. Secondly, the lack of specialized tools prevents citizens from making precise assessments and reliable predictions regarding the meteorological evolution of the phenomenon. Conversely, information provided by authorities based on meteorological forecasts and specialized data represents a more reliable and accurate source. The experts behind these forecasts can accurately interpret complex data and provide a professional assessment of the risk. Therefore, while direct observation can provide a general perception, the guidance of authorities offers a more in-depth and reliable evaluation of the hydrogeological risk level. It is important to note that, despite their precision, even the latter methods have margins of error.

### 4.3. Citizens’ Description

Each citizen is characterized by their ability to see and understand the phenomena. Citizens have a certain degree of skill in estimating events. Since these events are probabilistic, citizens may correctly identify the event in some situations but not in others. Therefore, citizens’ ability is modeled in probabilistic terms. Hence, for the same event and with the same level of ability, some citizens will identify it correctly, while others will not. To represent these abilities, we associated with the citizens’ evaluations a standard deviation related to the meteorological events. The citizens also have a trust value for each of their information sources.

Further, the citizens possess an initial monetary capital; they want to save it, but it could decrease in time. Each citizen decides whether to invest their capital to make security modifications to their property, reducing or even nullifying the possible damage in case of an event. If they do not, they expose themselves to the risk of a high level of damage.

### 4.4. The Authority

Concerning the authority, it aims to inform citizens about what will happen and to encourage them to invest to reduce possible damages. Its ability to produce forecasts is modeled through a standard deviation. Similarly to the citizens, even the authority has capital, which can be used to encourage citizens to take preventive measures for incoming events. In particular, we modeled three possible strategies:

1. *Punitive authority*: it fines citizens if it asks them to take measures and they do not. However, it will not discover all the guilty citizens but just a percentage of them. The fine value is 1, so it is equal to the maximal investment, and the fine probability is 20%. The fines increase the capital of the authority.
2. *Encouraging authority*: it monetarily helps citizens take measures; if the citizens invest, they will receive an incentive equal to 50% of the investment.
3. *Punitive and encouraging authority*: it fines citizens if they do not take measures, and it helps them to take measures.

In addition to this, the authority suffers damage because of the wrong choices of citizens. In fact, when the population is affected by a medium or critical event, it will be necessary to help it: the cost of hospitalization for the wounded, the cost of restructuring infrastructure, the cost of helping the population, etc. All these necessary maneuvers of authority will reduce its capital.

### 4.5. How Citizens Decide

Natural risk analysis can be carried out by leveraging qualitative data and predictive models. This is typically what experts do, but common people do not have access to these sophisticated approaches and require a practical way to perceive the risk.

As mentioned earlier, we modeled citizens using cognitive agents. These cognitive agents want to identify the most convenient choice in every situation, which means minimizing the cost of their actions and the risk they are subjected to. These agents think, reason, and interact to determine what actions to take in their specific situations. In this regard, they need to subjectively assess the risk they are facing.

As highlighted in the literature [68], probabilistic risk assessment computes the risk as a function of the following:

1. The *magnitude* of the event;
2. How likely it is that the event occurs, i.e., its *probability of happening*.

The agents do not know the actual risk to which they are subjected, so they try to estimate it. First, they need to compute the event’s *probability of happening*. This can be performed by considering the distribution of past events or gathering information from the information sources. From the GPDF described in Section 3, the citizens can subjectively estimate the probability with which each event will occur: P(e1), P(e2), and P(e3).

After that, they can quantify the damage they may suffer, which also depends on their actions. They could decide to do one of the following:

1. To make a *maximal investment*: in this case, the damage is reduced to zero with light and medium events, and it is minimal with critical events. This is the most conservative choice.
2. To make a *medium investment*: in this case, there is no damage from light and medium events, but there is important damage from critical events.
3. *Not to invest*: this decision saves money, but it exposes the citizens to high-level damages in cases of medium events and very high-level damages in cases of critical events.

Resuming the previous considerations, the factors that can influence citizens’ decisions are as follows:

1. The probability that each specific event occurs;
2. The costs related to each decision;
3. The estimation of the damages that the impending event could potentially cause.

Considering all this information, the probabilistic costs associated with individual decisions can be modeled as Equations (7)–(9), respectively, for the decision to make a maximal investment, to make a medium investment, or not to invest at all.

(7) $$\begin{array}{c} \begin{array}{cc} TotalCostMaxInvestment= & MaxInvestment+\\ \; & \frac{MaxDamage}{4}*P\left(e3\right)-Incentive \end{array} \end{array}$$

(8) $$\begin{array}{c} \begin{array}{cc} TotalCostMedInvestment= & \frac{MaxInvestment}{2}+\frac{MaxDamage}{2}*P\left(e3\right)-\\ \; & \frac{Incentive}{2}+\frac{Fine}{2}*P\left(Fine\right) \end{array} \end{array}$$

(9) $$\begin{array}{c} \begin{array}{cc} TotalCostNoInvestment= & MaxDamage*P\left(e3\right)+\frac{MaxDamage}{2}*P\left(e2\right)+\\ \; & Fine*P(Fine) \end{array} \end{array}$$

In Equations (7)–(9), incentives and fines are considered according to the authority’s profile. As for the fine, it is considered just if the authority predicted a critical or medium event and indicated to the citizen to make a corresponding investment but the citizen did not do so.

Please note that, in the case of a critical event, an agent will incur damage even if it opted for the maximum investment. These formulas utilize the actual cost an agent has to pay based on its chosen action and the potential damage it could suffer, providing only a probabilistic estimate. The actual cost an agent pays is contingent on both their decision and the actual event that occurs.

Clearly, we have considered all the information that a rational agent could leverage in the case of natural hazards. However, it is not guaranteed that agents will act in this manner. Consider, for example, the concept of “bounded rationality” introduced by Parker [69]: it is not guaranteed that an individual in such a risk situation will act rationally. This is precisely why, in addition to fully rational agents, we also take into account the presence of agents with lower cognitive ability. In other words, these agents will only consider a portion of the available information, engaging in simpler reasoning. Therefore, we introduced five types of agents in the simulation:

1. *Random agents*: they do not consider any information about the event, their decision is absolutely random. This is the most basic kind of agent; thus, we used them as a sort of control group, as a reference for the other agents’ performance.
2. *A priori agents* (AP agents): this type of agent considers the a priori probabilities of the events that characterize the world but does not refer to the specific situation. They act considering the most probable event; therefore, they will always make the same choice.
3. *A priori and costs agents* (APC agents): they can evaluate both the a priori probabilities and the costs and damages corresponding to each decision. Although they produce a better evaluation compared to AP and random agents, their decisions are still context-independent, so they will always make the same choice.
4. *Exploiting sources agents* (ES agents): these agents produce an estimation of the probability that each event is likely to happen using the available information sources. Then they decide according to the most probable event.
5. *Exploiting source and costs agents* (ESC agents): this is the most possible rational agent in this context, as they take into account as much information as they can.

We care to highlight that, while the first four types of agents act intending to maximize the probability of correctly identifying the event, the fifth and final type acts with the goal of minimizing costs and potential losses. These two different strategies lead to different choices and performances.

### 4.6. Input Parameters

Several parameters on the platform can be customized to produce a multitude of possible scenarios.

The first thing that can be customized is the *events’ distribution*. Next, it is possible to change the *number of citizens* in the world, their *ability*, *initial capital*, and *profile* (random, AP, APC, ES, ESC). Concerning the authority, it is possible to set the *authority’s reliability*, its *profile* (punitive, encouraging, punitive and encouraging), and its *initial capital*. Concerning the decision costs and damages, it is possible to set the *costs* of the investments and the *damages* resulting from a wrong choice, as well as the *fine* amount, its *probability*, and the *incentives* provided by the authority.

### 4.7. Output Parameters

We are going to consider a few dimensions to understand the agents’ performance in each scenario. The most intuitive is the *final capital*. This provides a representation of how good the citizen is at saving money. This would be enough if the citizens’ capital was not influenced by the authority. Given that, we also need to take into consideration the citizen’s ability to avoid damage. This dimension is crucial since we do not just want them to save money, but to be safe. The *final capital* and *the avoided damage* need to be considered together as they provide a partial view of the performance if taken individually: an agent performs properly if it saves enough capital and if it avoids a high percentage of damage. For instance, if we live in a region where critical situations happen frequently, we will make a lot of preventive investments, but the invested capital will be justified by the high amount of avoided damage. If, on the other hand, we live in a region where risky weather events happen rarely, the investment will often be useless. These dimensions are available both to the citizens and the authority.

Another dimension is the *percentage of successes* the citizens have in facing a given type of event with adequate investment. Finally, we consider the *percentage of times the citizens follow what a specific source reports*. In particular, we will use it for the authority.

### 4.8. Workflow

The world consists of 32 × 32 patches, wrapping both horizontally and vertically. It is geographically divided into four equal-sized quadrants, with agents randomly distributed within each quadrant. Furthermore, each quadrant operates independently from a meteorological perspective.

During the simulation, agents gain experience with their information sources, assessing their reliability. At the beginning, the world contains an authority and a given number of citizens with a partial understanding of weather phenomena. To anticipate upcoming events, citizens begin collecting information from their respective sources. The authority provides forecasts, along with estimated criticality levels. It is important to note that these forecasts are not guaranteed to occur. The probability of forecast accuracy is tied to the precision of the authority, which depends on its standard deviation.

In addition to institutional information, the citizens can evaluate the situation on their own and can also exploit the evaluations produced by their neighbors by observing the effect of their decisions. As already said, the PDF of the social source is the result of the aggregation of the agents’ decisions in the neighborhood; if a neighbor has not decided, it is not considered.

Then, they estimate the events’ likelihood, considering all the information they can access and aggregating each single contribution according to how trustworthy the corresponding source is.

The citizens collecting information are marked as “thinking”, meaning that they have not decided yet. When they reach the decision phase, they must make a decision, which cannot then be changed. This information is then visible to the others (the neighborhood), who can in turn exploit it for their decision-making.

**Figure 1:**
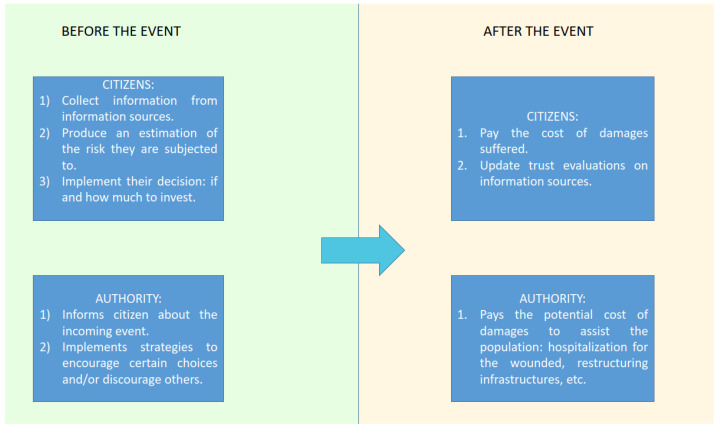
The flowchart of the simulation.

When the event ends, the citizens evaluate their sources’ performance, adjusting the corresponding trust values. After that, each citizen possesses a given capital and has avoided a given amount of damage; the same stands for the authority. The best strategy will maximize these two dimensions. Of course, it is not a given that the strategy that maximizes the citizens’ performance also maximizes the authority’s performance. Figure 1 reports the flowchart of the simulation.

## 5. Results

This section has the dual purpose of presenting the results of the agent-based simulation experiments, implementing what was described in the previous section on the NetLogo platform [67], and demonstrating the practical functioning of the system by providing a few examples considering the behavior of hypothetical populations.

As for the event distribution, we used data provided by [28] concerning Valtellina di Tirano: 77.91% *light event*; 17.44% *medium event*; 4.65% *critical event*.

We considered a small community of 200 citizens. We assume that they have no initial beliefs or prior knowledge biasing their choices, as we are not interested in investigating their effects in this context. At the beginning of the simulation, all citizens start with a neutral trust evaluation (0.5) for all their information sources. Therefore, through direct interaction with the sources and by observing what happens in the world, they estimate how trustworthy their sources are (see Section 3.1).

Concerning their ability to interpret phenomena, we consider a standard deviation of 0.7, which means that agents’ assessments will be correct in 45% of cases.

As for the authority, we assume that it is capable of informing all citizens with a high level of accuracy. Thus, it generates forecasts with a standard deviation of 0.5, indicating an accuracy rate of 70%. The choice of this parameter aims to make it more beneficial for citizens to refer to the authority rather than relying on their personal assessments. This choice is reasonable, since the authority generally has better resources and capabilities to provide more precise indications.

The values of the remaining parameters will be specified in the specific simulation settings since they vary from case to case. Each simulation lasts for 100 events, meaning that the citizens will be affected by 100 events, which is enough to properly judge their sources. Moreover, the results we report below represent the average values for 100 cases to eliminate the variability introduced in the individual runs.

### 5.1. First Simulation

With the first experiment, we aim to investigate the various profiles of citizens, starting from the simplest to the most sophisticated ones, and assess how they perform in the world. We use the following experimental settings:

1. Number of citizens: 200;
2. Citizens’ initial capital: 100 units;
3. Authority’s standard deviation: 0.5, which implies a correctness of 70%;
4. Authority’s profile: punitive, encouraging, punitive and encouraging;
5. Authority’s initial capital: 20,000 units;
6. Maximal investment: 1 unit;
7. Medium investment: 0.5 units;
8. Maximal damage: 10 units;
9. Medium damage: 5 units;
10. Fine amount: 1 unit;
11. Fine probability: 20%.

As for the initial capital, the absolute number is not fundamental. Instead, it serves as a unique reference point for comparing the different strategies of citizens. The authority’s capital is set to be equal to that of the entire community of citizens. The choice of the investment cost is designed to be much lower than the damage that the respective event would cause (a ratio of 1:10), ensuring that the upfront investment cost is always less favorable than investing based on probabilistic estimates. In other words, it is beneficial for citizens to stay informed about what is happening since the strategy of investing, while safe, is always very costly. The amount of the fine is equal to the maximum investment. This is because the fine is intended to discourage approaches that tend not to invest, making the option of not investing less attractive.

Table 1 and Table 2 report the values of the final capital and the avoided damage for all the types of agents we analyzed. Table 3 instead provides the sum of these two dimensions, which allows for a better interpretation of what is happening.

The simplest strategy to implement is the random one. Agents adopting it do not make any use of the information they have and simply make random decisions. In this case, they will correctly identify the event about to occur only once out of three, unnecessarily wasting their capital when there is no risk and exposing themselves to high risk without investing. Their final capital is very low, reaching even below zero if we consider a punitive authority (−8.49), and the avoided damage is very low as well, with a lower value of 77.22 in the P and E case.

A second type of agent is the AP agent, who evaluates the situation with a priori information, meaning they base their decisions solely on the event distribution in the world. Consequently, they react to the most likely event to occur, which in this case is the light event. As a result, they never invest and always incur all the damages. Although they consider initial information, their performance is even worse than that of the random agents. Their capital reaches a very negative value, as low as −38.91 in the P and E case, while their avoided damage is always equal to zero since they do not make investments.

When agents begin to consider the costs and damages associated with their decisions (the APC, a priori with costs agents), their performance starts to improve significantly. Although they do not utilize their information sources, they are aware that each decision is associated with a cost as well as a risk and potential damage. Taking this knowledge into account allows them to achieve higher performance. Indeed, as we can see in Table 3, the average overall improvement compared to random agents goes from 67.82% in the encouraging case to 98% in the punitive case.

Differently from APC agents, ES agents exploit just the information from their sources, but they ignore costs and damages. Their strategy aims to maximize the probability of guessing the event. Thanks to this, they obtain high performance, both in terms of final capital and avoided damage. The final kind of agent, the most complete one, is the ESC agent. This type of agent acts in a purely rational manner, utilizing all available information with the strategy of minimizing risk and the extent of damage they experience. In this case, the average improvement concerning random agents goes from 71.67% in the case of encouraging authority up to 119.74% in the case of punitive authority.

The performance of ES and ESC agents is very similar. The former have a higher final capital, while the latter have a higher avoided damage. Comparing the sum of these two dimensions (Table 4), it is clear that the latter perform better. As we can see from Table 3, the average improvement compared to random agents goes from 79.55% in the case of encouraging to 118.67% in the case of punitive authority.

These results clearly demonstrate that the superior decision-making strategy belongs to ESC agents (on the right), although ES agents achieve very similar outcomes. It is important to note that the performance gap between them would widen in the presence of more substantial damage. Additionally, it is essential to clarify that this analysis considers only direct damages and excludes indirect or secondary ones. The latter would further disadvantage ES agents’ performance, as they are more susceptible to critical events.

A peculiarity of ESC agents concerns the way they use their information sources. Normally, we expect them to follow the indications of the sources they consider most reliable. This is the case of ES agents, but ESC agents reason differently: it happens that, even if they highly trust a source (here, in particular, we are interested in the institutional source), they could not follow its indications. Although this phenomenon may appear unusual, it becomes natural when we consider their goals and thought processes. ES agents aim to maximize the probability of correctly predicting an event, leading them to rely on the most reliable sources. In contrast, ESC agents aim to minimize risk to reduce potential damages. Consequently, even if they have a high level of trust in a source, they may make different decisions from what the source suggests, as this often serves to reduce potential risks.

In this connection, we can see from Table 5 that ES agents follow the authority’s instructions in 99% of the cases. This is precisely because the authority is the best source they have, as it is the one communicating the most accurate information. On the contrary, ESC agents follow its indication just 58% of the time when the authority is punitive and 42% when it is encouraging or punitive and encouraging. This phenomenon is completely independent of trust values, which derive from the authority’s estimated reliability. Its average value is about 70%, and it is correctly identified in both cases. However, even though their trust evaluation of the authority is exactly equal, ES agents completely rely on the authority, as it allows them to maximize the results of their strategy, while the ESC agents go beyond what the source reports. Although ESC agents believe that the authority provides accurate information, they also consider the potential risks associated with making an incorrect decision. In other words, “I trust enough what you say, but since the risk is too high I would adopt precautionary behavior”. This is why they act differently. We call this phenomenon *“dissonance between evaluation and action”*.

After reviewing the strategies of citizens, let us now analyze the behavior of the authority. In particular, we consider its interaction with ES and ESC agents, which exhibit the most interesting characteristics. As already said, the authority has a strong influence on ES agents, who completely follow its indications. This is independent of the authority’s strategy to stimulate their action.

With the ESC agents, it is a very different story. They follow the authority’s instruction 58% of the time with a punitive authority and 42% of the time when it is encouraging or punitive and encouraging. These lower values should not be interpreted as a bad result; what happens here is that, thanks to the available incentives, they can afford to avoid much more risk, so they invest even if it is not strictly necessary. This allows them to avoid a critical event 10% more often than ES agents (the difference in the medium events is very low, as they already avoid the majority of them). On the contrary, fines are almost unused; they do not have any effect on these agents since, due to their very nature, they already tend towards protecting themselves.

We report the data on the authority’s final capital (Table 6) and the percentage of correctly identified critical events (*CICE*) by the ESC agents (Table 7).

The punitive authority can retain almost all of its capital; however, this significantly affects the performance of the agents. There are no significant differences between the encouraging and punitive and encouraging approaches since fines have a relatively minor impact in this context. To enhance their influence on the population, it would be necessary to increase the fine. In conclusion, for the purpose of maximizing citizens’ safety, the most effective authority profile is the encouraging one.

### 5.2. Second Simulation

As we have seen in the first experiment, the social utility of fines is very low. The agents think and act according to their personal well-being and this leads them to underestimate what the authority said and to take further preventive measures. However, in the real world, there can be situations in which the actions of the individual agents are non-independent but have ramifications for others. An example of such a situation could be complex residential structures, such as apartment buildings. In this case, the decision to take preventive measures benefits not only the individual agent but also their neighborhood. Therefore, the choice not to invest negatively affects not only the individual but also their neighbors. In other words, the citizens’ choices are interdependent. Of course, in such a complex social structure, a citizen could decide to exploit their neighbors by not investing and relying on others to do so for them (a free rider). We therefore introduce new calculation procedures for the agents—Equations (10)–(12)—to take into account the actions of their neighbors as well.

(10) $$\begin{array}{c} TotalCostMaxInvestment=\\ MaxInvestment+\frac{MaxDamage}{4*(nOfNeighbors+1)}*P\left(e3\right)-Incentive+\frac{CDFN}{4*P(e3)} \end{array}$$

(11) $$\begin{array}{c} TotalCostMedInvestment=\frac{MaxInvestment}{2}+\\ \frac{MaxDamage}{2*(nOfNeighbors+1)}*P\left(e3\right)-\frac{Incentive}{2}+\frac{Fine}{2}*P\left(Fine\right)+\frac{CDFN}{2*P(e3)} \end{array}$$

(12) $$\begin{array}{c} TotalCostNoInvestment=\frac{MaxDamage}{\left(nOfNeighbors+1\right)}*P\left(e3\right)+\\ \;\;\;\;\;\;\;\;\frac{MaxDamage}{2}*P\left(e2\right)+Fine*P\left(Fine\right)+\frac{CDFN}{P(e3)}+\frac{MDFN}{P(e2)} \end{array}$$

(13) $$\begin{array}{c} CDFN=\frac{MaxDamage}{nOfNeighbors+1}*nOfNeighbors*\left(1-socialTrust\right) \end{array}$$

(14) $$\begin{array}{c} MDFN=\frac{MaxDamage}{2*(nOfNeighbors+1)}*nOfNeighbors*\left(1-socialTrust\right) \end{array}$$

In Equations (13) and (14), CDFN and MDFN are, respectively, the critical and medium damage expected for a citizen to receive from neighbors. The citizens’ choices are influenced by what their neighbors do, as each choice implies possible further damage that they cannot avoid but can reduce. To estimate the potential damage from the neighbors, we use the dimension of social trust: the more I believe that my neighbors’ decisions are correct, the less damage I expect from them, and vice versa. So, the effect of weather events on the agents remains the same as in the first experiment. What changes in this case is that the impact of this damage no longer depends on the individual choice of the citizen but is now mainly dependent on the choices of their neighbors.

In this experiment, we will only consider ESC agents so as to fully apply the reasoning model introduced above. Thus, we report below (Table 8) what happens to ESC citizens. As we can see, it immediately stands out that these agents make a lower number of maximal investments compared to the previous experiment and instead focus on medium investments. This phenomenon occurs because, since they can trust their neighbors’ choices, they estimate that they will receive less damage from the events. Therefore, they can afford to concentrate on smaller investments and decide to make use of maximal investments only when they believe it is really necessary.

In other words, in this case, the neighborhood functions as a *social absorber* of risk. It should be noted that this does not mean that the citizens will transfer their damage to their neighbors, but rather that, thanks to social interaction, the risk they are exposed to is reduced. In fact, personal risk becomes a collective dimension that is shared with neighbors. If there is a high level of community trust, this mechanism can prove to be a valuable resource, allowing citizens to achieve high performance as they will preserve their own capital. Remarkably, the results show that they perform even better here than in the first experiment.

However, it is still true that the very nature of this social structure could potentially encourage unfair behavior, as someone may attempt to exploit this positive attribute for personal gain. In the subsequent part of this scenario, we introduce a certain percentage of *free rider* agents into the system who aim to offset their personal losses by relying on the neighborhood without making any contribution. Specifically, we investigate a scenario with 20% of the agents being free riders.

Evaluating the final capital and the avoided damage (Table 9 and Table 10), it is clear that the presence of free riders poses a problem for the entire social system. First and foremost, they incur significant harm. Although they save money from the investment, they experience greater damage both directly from the event and from their neighbors. Moreover, these agents harm the ESC agents.

In the first part of this experiment, we were able to harness the positive effect of the social structure thanks to the high level of social trust. However, when we introduced free riders attempting to exploit their neighbors’ benevolence, the ESC agents needed to react. However, in doing so, they lost the opportunity to benefit from their neighborhood trust, which instead became a social burden they had to sustain. The logical consequence here is an increase in investments, both medium and maximal. As a result, despite the measures implemented to mitigate this problem, their performance is slightly penalized as well.

As for the authority, from the results (Table 11), it emerges that the social risk absorption effect allows for a higher impact of its indications on ESC decisions. Indeed, in this scenario, both the encouraging and punitive and encouraging authorities manage to save more capital units (see Table 12). This result occurs because agents have the opportunity to take a higher level of risk. Therefore, they will limit their investments (and hence request fewer incentives from the authority) only when it is truly necessary. However, this also implies that, in the event of a critical event that was not correctly anticipated, agents will be less capable of addressing this event. It should be noted that medium events are addressed with the correct investment in 91% of cases when the authority is punitive and in 95% of cases when it is encouraging or punitive and encouraging. Regarding critical events (Table 13), as for the first experiment, the encouraging strategy has a greater impact than the punitive one. However, the best performance is achieved with the punitive and encouraging strategy. This time, in fact, since agents tend to be conservative in their investments, fines have a greater impact on their decisions.

## 6. Discussion

In this study, we introduced a social simulation platform designed to investigate the social behavior of a community in the presence of hydrogeological phenomena with varying degrees of criticality.

For this simulation, we chose to make use of multi-agent systems due to their aptitude for exploring community phenomena that arise from the behavior of individual agents [70,71]. Additionally, their capacity to model cognitive agents in depth allows us to gain insights into human behavior and reasoning. In our model, citizens make decisions based on their subjective risk perception, relying on quantitative factors such as the potential damage they might incur and the subjective estimation that the event will occur. Additionally, this modeling approach allows us to explore the connection between risk perception and the information sources that convey risk-related information.

In these scenarios, we have citizens on one hand and authorities on the other. As we specified, the interests of these two entities do not always align, as they have at least partially distinct objectives. Consequently, we conducted an analysis of optimal strategies within different contextual settings.

We introduced two distinct scenarios, each marked by unique social dynamics. In the first scenario, citizens’ decisions are entirely independent, while in the second scenario, we explored a situation where they mutually influence one another.

Concerning citizens, we modeled their choices to be strictly dependent on their subjective risk perception [72,73]. If authorities wish to intervene in their decisions, it is precisely this perception that needs to be modified. To a lesser extent, interventions can also be made through fines and incentives [74].

We examined various cognitive strategies for the citizens based on the amount of information they consider. While utilizing all available information ensures the best performance, there are situations, especially in high-risk contexts, where it may be challenging to consider all available knowledge.

In this regard, results suggest that relying solely on information sources can yield good results, even when ignoring cost and potential damage considerations. This could be a consequence of the fact that even the most delicate decision-making processes can be simplified when relying on clear and easily understandable sources of information [75,76]. If the population is not inclined to make completely rational decisions, simplifying the decision-making process can lead to more effective results. In addition, risk perception is often subjective and can vary among individuals. If authorities present accurate and accessible information, the population may respond positively, even without a detailed assessment of costs and damages. This consideration in itself provides valuable insights for authorities. A more advanced strategy would involve not just focusing on the information but emphasizing the utilization of specific information over others. For example, if the population is not inclined towards fully rational decision-making (as not all members can always consider all relevant parameters), it may be more effective to encourage the use of information sources rather than focusing on providing detailed information about costs and damages.

Another remarkable outcome of our study is the phenomenon we termed *“dissonance between evaluation and action”*, which was specifically observed in ESC agents, i.e., agents relying on both information sources and cost considerations in their decision-making process. Despite placing a high level of trust in their information sources, their ultimate decisions diverged from the information reported. This divergence arose from their engagement in reasoning at a distinct level of abstraction, wherein they accounted for potential risks associated with an incorrect choice. This phenomenon highlights the intricate nature of decision-making, especially in the context of probabilistic forecasts. Our findings underscore the importance of acknowledging even less probable events, as these events, if realized, could have a significant impact. The dissonance observed in ESC agents emphasizes the nuanced interplay between trust in information sources, the incorporation of cost considerations, and the cognitive processes involved in decision-making. Effectively addressing this dissonance requires a deeper understanding of how individuals balance these factors and the cognitive strategies they employ when faced with hydrogeological risk scenarios.

Another intriguing phenomenon that emerged in the second experiment concerns the use of the social structure as a *social absorber of risk*. Indeed, this structure represents a shared resource that agents can exploit and rely upon to mitigate the individual risks they face. The validity of this result is highlighted by its ability to demonstrate how cooperation and resource-sharing within a community can significantly contribute to the mitigation of individual risks. In this way, citizens can afford to undergo greater risk while making fewer individual investments. The possibility of relying on this phenomenon allows agents to achieve better performance, even compared to agents in the first scenario. Clearly, this social effect has a significantly positive impact on both individuals and the community. This aspect is crucial as it suggests that social cooperation can translate into tangible benefits for the entire community, enhancing overall performance in the management of hydrogeological risks. The impact of this result is relevant as it provides authorities and emergency managers with a strategic opportunity. The awareness that a well-organized social structure can act as a buffer for risks suggests that promoting collaboration within communities can be equally important as providing detailed information about costs and damages. Furthermore, it underscores the need to encourage social practices and cooperative behaviors as integral parts of risk management strategies.

However, this phenomenon also opens the door to potential side effects. Indeed, malicious and uncooperative agents (free riders) could decide to exploit the social structure for their personal advantage by not making any investments and shifting the burden onto others. In fact, the experiments reveal that this is indeed a deleterious strategy, even for the agents implementing it, as the social structure ceases to function as an absorber and instead becomes a social burden. In response to this, the remaining agents will increase the number of investments to mitigate the damage caused by their free-riding neighbors.

Concerning the authority, we analyzed three possible strategies aimed at encouraging citizens to adopt appropriate behaviors. From the results, it emerges that, in general, an encouraging authority has a greater impact than a punitive one. The strategy of fining has a stronger effect in the second scenario, where citizens’ choices are strongly interdependent. In this scenario, the authority maximizes its performance by implementing a punitive and encouraging strategy.

The purpose of this work is to provide a framework that can be instantiated across multiple contexts and is not specifically focused on a particular population, city, or region. The platform is also flexible regarding information sources, which may vary in number, trustworthiness, and type. Yet, the premises of the social structure must remain consistent: a society in which citizens have capital to protect themselves and there is a central authority with the goal of preserving the population and the means to act toward that end.

In this regard, a case of particular interest involves third-world countries where the rate of precipitation is low. In this specific scenario, some unique challenges may emerge. For instance, on the one hand, there may be a lack of trust in what authorities communicate. From this perspective, implementing better communication strategies and building a trusting relationship with the population could be a more effective path to pursue. On the other hand, it should also be emphasized that, in many of these countries, citizens’ primary issue is the lack of resources to make investments. In these cases, local authorities should not simply focus on encouraging investments in security but could instead become active participants in the investments, directing their efforts towards the direct management of social security, which is undoubtedly a complex task.

## 7. Conclusions

The results presented in this study, along with the implemented platform, provide valuable insights and a solid starting point for authorities and emergency managers. This is because, in addition to identifying the most effective strategies in the various situations we have considered, they enable an understanding of the effects and connections that arise within the population. This work contributes to aiding hydrogeological risk management in several ways. First and foremost, it allows us to investigate decision-making processes in the context of hydrogeological risk. The work presents a model that takes into account social behavior and citizens’ decisions in hydrogeological hazard situations. This can be valuable in understanding how individuals respond to varying levels of hydrogeological risk events. The information obtained can assist authorities in developing strategies to positively influence people’s behavior and promote mitigation actions.

Indeed, the work aims to investigate the role and impact of institutional communication. The article examines various strategies that authorities can employ to influence citizen behavior. For instance, it emphasizes that an encouraging approach by authorities tends to be more effective than a punitive one, which can serve as a significant input for policy formulation.

Furthermore, the article examines the effects of the social structure on risk sharing. We highlighted how the social structure can act as a “social absorber of risk”, suggesting that the sharing of resources and cooperation among individuals and communities can contribute to reducing individual environmental risks.

Last but not least, the article highlights the role of institutional trust in this context, emphasizing the importance of citizens’ trust in authorities. Trust is crucial for the actions of authorities to have a direct impact on the safety and well-being of the population. The effectiveness of these strategies is closely influenced by citizens’ perceptions of authorities. If people have trust in the authorities, they are more likely to respond positively to their directives and incentives.

Of course, this study is not without limitations. Firstly, we did not address the investigation of the effect of individuals’ impulsive and irrational decisions in these risk contexts. This could involve exploring psychological factors, behavioral biases, and emotional responses that may significantly affect decision outcomes. Although this aspect has been partially explored in other studies [77,78,79], it requires a more careful analysis. Additionally, the impact of fines should be further investigated and considered in varying amounts. In fact, we have observed that, in some scenarios, the weight of fines was relatively less significant compared to other factors influencing individuals’ decisions. Analyzing how different penalty levels influence citizens’ decision-making processes will contribute to the development of more targeted and impactful regulatory strategies. This task may involve experiments or simulations with diverse fine scenarios to identify optimal punitive measures for encouraging desirable behaviors. To broaden the applicability of our results, future tasks should focus on adapting the findings to regions with fewer data sources. Indeed, the model employed in this work for source management is robust in this regard. It assesses the information that agents possess based on the estimated reliability of the sources, regardless of their quantity. Nevertheless, this also involves developing strategies that are robust in the face of data scarcity. We reserve the possibility of exploring such aspects in greater detail in future studies.

## Figures and Tables

**Table 1:** *Final capital* of random, AP, APC, ES, and ESC agents by type of authority.

	Punitive	Encouraging	P and E
Random	−8.49	19.07	17.07
AP	−38.34	−33.88	−38.91
APC	26.35	51.59	51.92
ES	47.31	59.57	59.46
ESC	36.92	54.67	54.9

**Table 2:** *Avoided damage* of random, AP, APC, ES, and ESC agents by type of authority.

	Punitive	Encouraging	P and E
Random	77.64	77.75	77.22
AP	0	0	0
APC	110.56	110.89	110.11
ES	104.64	106.64	105.35
ESC	116.29	119.17	117.77

**Table 3:** *Final capital* plus *avoided damage* of random, AP, APC, ES, and ESC agents by type of authority.

	Punitive	Encouraging	P and E
Random	69.15	96.82	94.29
AP	−38.34	−33.88	−38.91
APC	136.91	162.48	162.03
ES	151.95	166.21	164.81
ESC	153.21	173.84	172.67

**Table 4:** The sum of *final capital* and *avoided damage* for ES and ESC agents by type of authority.

	Punitive	Encouraging	P and E
ES	151.95	166.21	164.82
ESC	153.21	173.84	172.67

**Table 5:** Percentage of times in which ES and ESC agents follow the authority’s indications.

	Punitive	Encouraging	P and E
ES	99.26	99.28	99.07
ESC	57.62	42.15	42.19

**Table 6:** *Final capital* of the authority.

	Punitive	Encouraging	P and E
Final Capital	18,382.22	12,337.77	12,354.86

**Table 7:** Percentage of correctly identified critical events (*CICE*) by the ESC agents.

	Punitive	Encouraging	P and E
*CICE*	72.67	82.45	82.1

**Table 8:** *Final capital* and *avoided damage* of ESC agents.

	Punitive	Encouraging	P and E
Final capital	51.48	68.23	68.44
Avoided damage	120.84	122.94	124.65

**Table 9:** *Final capital* of ESC and free rider agents by type of authority when 20% are free riders.

	Punitive	Encouraging	P and E
ESC	43.29	58.87	58.96
Free riders	46.44	54.51	50.09

**Table 10:** *Avoided damage* of ESC and free rider agents by type of authority when 20% are free riders.

	Punitive	Encouraging	P and E
ESC	111.57	116.25	116.4
Free riders	87.94	91.56	91.68

**Table 11:** Percentage of times in which ESC agents follow the authority indications.

	Punitive	Encouraging	P and E
ESC	83.43	68.85	70.14

**Table 12:** *Final capital* of the authority.

	Punitive	Encouraging	P and E
Final Capital	18,435.84	14,772.86	14811.06

**Table 13:** Percentage of correctly identified critical events (*CICE*) by the ESC agents.

	Punitive	Encouraging	P and E
*CICE*	58.55	68.68	71.83

## Data Availability

No new data were created or analyzed in this study. Data sharing is not applicable to this article.

## Abbreviations

The following abbreviations are used in this manuscript:

PDF	Probability density function
GPDF	Global probability density function
GE	Global evidence
NF	Normalization factor
AP	A priori agents
APC	A priori and costs agents
ES	Exploiting sources agents
ESC	Exploiting sources and costs agents
P and E	Punitive and encouraging authority
CDFN	Critical damage expected to receive from neighbors
MDFN	Medium damage expected to receive from neighbors
